# Sanitation and the Panama Canal

**Published:** 1905-09-16

**Authors:** 


### SANITATION AND THE PANAMA CANAL.

The range and value of sanitary science in its application to practical affairs is a text which in modern days serves as the motive for many sermons. Indeed, so frequent are the effusions to which it gives rise that iteration has somewhat dulled the ear, and neither the statement of hygienic laws nor the penalties which attach to the breach of them can expect to prove an attractive topic to a popular audience. The plain man conducts his business and takes his recreation free from any keen recognition of the truth that both one and the other would promptly be rendered impossible were a numerous and active hygienic army to cease its efforts to restrain and control a whole host of subtle and dangerous enemies. But where the lecturer and writer on sanitation may fail, and where bacteriology, even when illustrated by lantern slides, may produce but a transitory and fleeting impression, the true state of affairs may possibly be conveyed through the medium of an object-lesson. Such a lesson, for those who care to study it, may readily be found in recent proceedings in connection with the construction of the Panama Canal. The enormous economic importance of that undertaking is probably imperfectly appreciated even by statesmen and experts, and stirs hardly at all the thought of the ordinary individual. But almost everyone can understand that the scheme involves vast engineering difficulties, and that its completion will mean trading developments of great significance to several nations, and, among these, to the peoples of the United Kingdom. Comparatively recent history has shown how serious in a financial and national sense may be the failure of such a project, and what disastrous consequences a collapse may entail. But with a prospect of failure no responsible person now troubles himself. The Great Republic of the West, under the guidance of its strenuous President, will refuse to know the meaning of any such word, and successful attainment may be taken for granted. It is here, indeed, that we find the point of immediate interest, for it has recently become obvious that in advance of the Canal, and of the splendid prospects to which it is to lead, there is a problem in practical sanitation which demands solution, and the neglect of which would inevitably bring all engineering schemes and plans to an abrupt and melancholy conclusion. The earlier plans for the construction of the Canal had the business-like sound and aspect generally associated with the nation responsible for their inspiration. They represented the national impatience as well as the national thoroughness. There were, it is true, to be some systematic efforts at practical sanitation in the cities of Panama and Colon and also along the entire route to be taken by the Canal. But the chief business of the entire undertaking was not to pause for these, and, as a matter of fact, it did not pause. Steam shovels of maximum capacity and vast armies of labourers were promptly at work and the determination of everybody concerned was, in the President's characteristic phrase, "to make the dirt fly." Now all this process of actual canal making has been forced to call a halt. Bacteriological forces and epidemic influences have proved too much even for the all-pervading energy of the American engineer. Malaria, dengue, typhoid, and, above all, yellow fever, have shown themselves masters of the situation. The deaths from these diseases and the demoralisation so induced have compelled the authorities entirely to alter their plans. All works immediately concerned with the construction of the Canal have been abandoned and the activities of the army of engineers and labourers are now directed to the sanitary measures necessary to make the country approximately free from epidemic disease. When this has been accomplished no doubt the word will once more be "make the dirt fly," but in the meantime engineering and national ambitions are postponed by the claims of sanitation. A more impressive exhibition of the relationship of sanitary science to severely practical undertakings could hardly be imagined. It has its significance to every member of every civilised community. But, in addition, it may claim the special attention of a nation which has large concerns and undertakings in tropical and sub-tropical latitudes, and whose sons, in civil and military enterprises, find themselves in all the ends of the earth. *Sanitas, sanitatum, omnia sanitas* has a field of application so wide that even transatlantic resolution has had to bow to its behest.

